# Self-Esteem as a Mediator between Personality Traits and Body Esteem: Path Analyses across Gender and Race/Ethnicity

**DOI:** 10.1371/journal.pone.0112086

**Published:** 2014-11-06

**Authors:** Małgorzata Skorek, Anna V. Song, Yarrow Dunham

**Affiliations:** 1 Psychological Sciences, University of California Merced, Merced, California, United States of America; 2 Department of Psychology, Yale University, New Haven, Connecticut, United States of America; University of Udine, Italy

## Abstract

Prior literature examines the *direct* relationship between personality traits and body esteem. This article explores the possibility that self-esteem mediates this relationship. 165 undergraduate women and 133 men (age 18–21; 42.6% Hispanic, 28.9% Asian, 28.5% Caucasian) completed items measuring personality traits (Big Five), self-esteem, and body esteem. Path analyses were used to test for mediation. The analyses confirmed that in both men and women self-esteem mediated the relationship between three personality traits and body esteem: higher levels of conscientiousness, emotional stability, and extraversion were associated with higher self-esteem and consequently higher body esteem. Once self-esteem was included in the model the relationships between personality traits and body esteem were not significant, suggesting full mediation. In addition, the analyses revealed several racial/ethnic differences. In Asian American participants, self-esteem mediated the relationship between conscientiousness and body esteem and between emotional stability and body esteem. In Hispanic Americans, self-esteem mediated the relationship between conscientiousness and body esteem and between extraversion and body esteem. And in Caucasian Americans, self-esteem mediated the relationship between emotional stability and body esteem and between extraversion and body esteem. The most important contribution of this study is evidence for an *indirect* relationship between personality traits and body esteem, with this relationship being mediated by self-esteem. This has important implications for the study of personality and eating disorders in young adults, most particularly implying a need for more emphasis on self-esteem as a predictor of body image problems.

## Introduction

Adolescents and young adults are subject to heavy pressures from their peers, parents, and mass media to meet appearance standards [1], [2]. As a result, young men and women are often dissatisfied with their bodies [3], [4]. Such negative body image puts them at severe health risk because it strongly predicts the development of disordered eating [5] and depression [6], [7], both of which can persist into adulthood. Several researchers have speculated that personality traits affect men and women’s body esteem and have investigated correlations between them (e.g., [8]), but the exact nature and directionality of the relationship between personality traits and body image is still unknown, a critical stumbling block for identifying and intervening on vulnerable populations (e.g., young adults susceptible to developing eating disorders). Specifically, elucidating the more precise nature of the relationship between personality and body image will help practitioners better screen and treat these disorders.

Body image is a broad construct that relates to a person’s perceptions, feelings, and thoughts about his or her own body [9]. This construct has been operationalized in many different ways, for example as body esteem, body dissatisfaction, body distortion, body appreciation, or drive for thinness and muscularity [10]. Body esteem and body dissatisfaction are very similar constructs, differing primarily in the specific questions used in their respective survey instruments (body dissatisfaction scales ask about satisfaction or dissatisfaction with different body parts, whereas body esteem scales focus on positive or negative feelings towards specific body parts; [10]). Body esteem, body dissatisfaction and other similar body image concepts are often used interchangeably [10]. Here we focus on body esteem, which is one of the most general and oft-studied aspects of body image, and has served past researchers well as an index of body dissatisfaction more broadly (e.g., [11]).

Prior studies have established a link between personality traits and body esteem. In particular, prior work has revealed a negative relationship between emotional stability (often referred to as neuroticism when focusing on the opposite end of the spectrum) and body dissatisfaction in both women [12]–[14] and men [15]. Moreover, emotional stability and extraversion predicted more positive body esteem in men and women [8], [16]. Experimental studies suggest that emotional stability moderates the effect of exposure to idealized images of women on body esteem, with women having low emotional stability (i.e., high level of neuroticism) experiencing lower body esteem after exposure to such images [17], [18]. Finally, research in clinical populations has established a link between personality traits and personality disorders and the onset, symptomatic expression, and maintenance of eating disorders (for a review, see [19]). The relationships between personality traits and negative body image may be explained by the fact that individuals who have low emotional stability are more emotionally reactive to social comparisons and generally more negative towards themselves and their appearance [16]. By contrast, extraverts are generally more outgoing and positive in affect, leading to more positive self-evaluations [16].

As described above, prior work has to date focused primarily on the direct relationship between personality traits and body esteem. However, personality traits relate to other individual difference factors that are in turn related to body esteem, raising the possibility of a more complex relationship. Specifically, according to McCrae and Costa’s five-factor theory [20], [21], the Big Five personality dimensions (agreeableness, conscientiousness, emotional stability, extraversion, and openness to experience) influence people’s self-conceptions including trait self-esteem. An individual’s self-conception is assumed to be selectively represented in ways that are consistent with that person’s personality traits thereby giving a sense of coherence to the individual [21]. This consistency between personality traits and self-esteem is supported by work showing that Big Five personality dimensions account for 34% of variance in self-esteem [22]. In particular, three traits are the most related to self-esteem: emotional stability (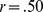), followed by extraversion (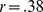), and conscientiousness (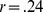; [22]). The correlation between the remaining personality traits (openness and agreeableness) and self-esteem is lower (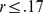 for both; [22]; see also [23]).

Self-esteem is also closely related to body esteem in both men and women [11], [24] and it is considered one of the strongest predictors of body esteem [25]. This relationship is hardly surprising; many have suggested that young men and women have been socialized to believe that appearance is a primary basis for self-evaluation and evaluation by others [10], [26], and low satisfaction with one’s body is very often associated with low self-esteem and vice versa [11], [27]. This relationship also falls out of Self-concept Theory, which proposes that dissatisfaction with a domain of great importance to one’s self (in this case one’s body) is more damaging to one’s self-esteem than dissatisfaction in domains of less importance [28]. This is evident in individuals with eating disorders, who, according to a prominent account of bulimia nervosa [29], judge themselves primarily in terms of their shape, weight, or eating habits. This over-evaluation of body and diet is the fundamental maintaining mechanism of their eating disorder [30]. Bearing out these contentions, the correlation between body satisfaction and self-esteem is remarkably strong, with an average correlation of.65 in the U.S. [31].

Although personality traits, self-esteem and body esteem are all closely related, the precise nature of how personality traits relate to the other two constructs is still unknown. Because personality traits (in particular conscientiousness, emotional stability, and extraversion) are correlated with self-esteem and self-esteem in turn is correlated with body esteem, we hypothesized that self-esteem mediates a broader-based relationship between personality traits and body esteem. Based on prior literature and the five-factor theory [21] described above, we developed the following more specific hypothesis:

*H1: Self-esteem mediates the relationship between three personality traits (conscientiousness, emotional stability, extraversion) and body esteem*.

We expected no mediational relationship between agreeableness and body esteem and between openness and body esteem because these two personality traits correlated less strongly with self-esteem [22]. Thus:

*H2: Self-esteem does not mediate the relationship between two personality traits (agreeableness, openness) and body esteem*.

In exploring the above hypotheses, we must note that the *direction* of the relationship between body esteem and self-esteem has not yet been established in the literature [25]. That is, there exists the possibility of an alternative model in which *body esteem* mediates the relationship between personality traits and self-esteem (instead of the other way around). We find the former more plausible because self-esteem, by definition a highly general self-appraisal, likely exerts influence on many more specific forms of self-appraisal (including appraisal of one’s own body), while the reverse pathway from a more specific to a more general form of self-appraisal would not be expected to broadly hold. We considered it essential to address this issue by formally testing both directional models. In testing our hypothesized model, we included all five personality traits, two of which (agreeableness and openness) have been rarely examined previously with regard to their relationship to body esteem and self-esteem; however, given evidence regarding which traits most reliably predict self-esteem, we expected relationships to emerge only for conscientiousness, emotional stability, and extraversion.

Another unsettled issue concerns gender differences in body esteem, with some studies reporting that women tend to exhibit lower body esteem than men [32]–[34] and others reporting no differences [11]. A meta-analytic review suggested small gender differences in body image, with men generally being more satisfied with their bodies than women [35]. While some studies found that women report lower self-esteem than men [36], some investigators have not found such gender differences [11], [32]. Meta-analyses of gender differences in self-esteem also confirmed a small difference favoring males [37], [38]. With regard to personality traits, a cross-cultural meta-analysis demonstrated that women scored higher than men on agreeableness, conscientiousness, and extraversion, and lower on emotional stability [39]. Given the complexity of these findings, we did not predict any specific gender differences, instead treating this part of our study as more exploratory.

Although research has rather consistently reported gender differences in body esteem, differences across race/ethnicity are less clear due to limited literature including diverse populations [3]. Nevertheless, many researchers have suggested that young men and women from different racial/ethnic backgrounds may vary in their body esteem because meanings of the body depend on cultural context [40]. Prior work suggested that body esteem of Caucasian Americans is lower than that of Hispanic American men and women [33] and Caucasian women’s body esteem is lower than that of Asian American women [41]; however, others report no racial/ethnic differences [42]. A recent meta-analysis demonstrated that whereas Asian American women had lower body dissatisfaction than Hispanic women, and African American women had lower body dissatisfaction than Caucasian American women, other racial/ethnic group comparisons were small and non-significant [3]. Unfortunately, no work on racial/ethnic differences in male body esteem exists to the authors’ knowledge.

Turning to self-esteem, there is meta-analytic work to suggest that Caucasian Americans have higher self-esteem than Asian and Hispanic Americans; and Asian Americans have lower self-esteem than their Hispanic counterparts [43]. Similarly, a recent meta-analysis of personality differences in five U.S. racial groups (Asian, Black, Hispanic, Caucasian, and Native American) found that Asian Americans are more agreeable and less emotionally stable than Caucasian Americans; no further sizeable differences were found for other group comparisons [44].

The preceding findings suggest the possibility of racial/ethnic differences in all three key constructs of interest (i.e., body esteem, self-esteem, and personality). However, the complex patterns again preclude strong hypotheses, leading us to again explore the hypothesized relationships across race/ethnicity.

In summary, the central goal of this article is to extend our understanding of young men’s and women’s body image by attempting to specify the pathway by which personality traits influence body esteem, and in particular the possibility that self-esteem plays a primary mediational role. The secondary goal is to explore whether the same patterns hold across gender and racial/ethnic groups, which have been woefully understudied in the past. Our results will shed light on the psychological mechanisms linking personality to body image, thereby contributing to efforts to accurately identify and intervene on susceptible populations.

## Materials and Methods

### Participants

One hundred and sixty five undergraduate women and 133 men (age range 18–21, 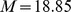, 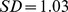) from a university in Central California (USA) volunteered to participate in the study in exchange for credit for their introductory psychology course requirement. One hundred twenty seven participants were Hispanic American (42.6%), 86 were Asian American (28.9%), and 85 were Caucasian (28.5%). This distribution reflects this university’s diverse student population. The study was approved as exempt by the Institutional Review Board of the University of California.

### Measures

#### Personality traits

We administered the Ten-Item Personality Inventory (TIPI; [45]) in which participants indicate how strongly they associate ten pairs of characteristics with the self using a 7-point Likert scale (1– strongly disagree, 7– strongly agree). For example, items included ‘extraverted, enthusiastic’ and ‘calm, emotionally stable’, with two pairs of adjectives corresponding to each ‘Big Five’ personality dimension: agreeableness, conscientiousness, emotional stability, extraversion, and openness to experience [21]. An average rating for each pair of adjectives for each dimension was used as the individual’s score on that dimension (scale 1–7). Even though the TIPI is a very short scale, it has been demonstrated to be adequate in terms of 1) convergence with widely used Big Five measures in self, observer, and peer reports, 2) test-retest reliability, 3) patterns of predicted external correlates, and 4) convergence between self and observer ratings [45]. Several other brief measures of personality traits exist, including the NEO Five-Factor Inventory (NEO-FFI; [46]) and the Single-Item Measure of Personality (SIMP; [47]), but the TIPI generally outperforms these other scales [48].

#### Self-esteem

To assess trait self-esteem we used Rosenberg’s Self Esteem Scale (RSES; [49]), a ten-item measure using a 4-point Likert scale (0– strongly disagree, 3– strongly agree) that includes five positive and five negative self-descriptive statements (e.g., “On the whole, I am satisfied with myself” or “I wish I could have more respect for myself”). The sum of the ratings assigned to each of 10 items, after reverse scoring the negatively worded items, indicated one’s self-esteem level (scale 0–30). Higher scores corresponded to higher self-esteem. Internal consistency of RSES in this study was good, 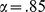 (women) and 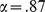 (men).

#### Body esteem

For the current study, we chose the Body Esteem Scale (BES; [50]) because it evaluates satisfaction with all body parts and functions and it is thus more specific than scales measuring general satisfaction with one’s body or few of its parts. Even though the BES is usually used to assess different body esteem subscales for men and women, all answers can be summed to form a single score reflecting participants’ overall body esteem [11], [32], [33]. Participants were given a list of 35 body parts and functions (e.g., lips, width of shoulders, body hair, agility) and were asked to indicate how they felt about each of them using a 5-point rating scale (1– have strong negative feelings, 5– have strong positive feelings). Participants’ overall body esteem score was calculated by adding up scores for all items (scale 35–175). The higher one’s summed score, the more positive their body esteem. The reliability and validity of the BES is presented in [50] and [51]. In our sample this scale achieved excellent internal consistency, 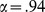 (women) and 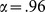 (men).

#### Body Mass Index (BMI)

BMI was included in the survey due to the fact that it may be influencing one’s body esteem. Participants were asked to report their weight and height, which were used to calculate their Body Mass Index (
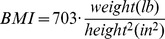
). This is a reliable way of measuring BMI; a recent meta-analysis demonstrated that self-reported weight differs only by 1–3.5% from individuals’ actual weight [52]

### Procedure

Participants signed up for the study using the university’s online recruitment system and were randomly assigned to one of two order conditions. In the first order condition, the measures appeared in the order as described above; in the latter, TIPI was followed by BES, and then RSES (demographic questions, including body height and weight, were asked at the end for all participants). Upon signing up participants received a link to an online survey and after an informed consent (written) they completed it individually in a non-laboratory setting. The study took less than 20 minutes to complete.

### Data analysis

We calculated descriptive statistics and zero-order correlations between variables (SPSS 17.0), and then undertook path analysis (MPlus 6.12), testing mediational relationships among variables using maximum likelihood to estimate parameters within the model. Model fit was assessed through Chi-Square goodness of fit, Root Mean Square Error of Approximation (RMSEA), Bentler Comparative Fit Index (CFI), and the Tucker-Lewis Index (TLI) [53]. All variables used in the study (five personality traits, self-esteem, and body esteem) have been previously validated and reached high scores of reliability, and were thus treated as observed rather than latent variables. This renders a path analysis approach identical to Structural Modeling (SEM); path analysis can be understood a special case of SEM in which only observed variables are employed in the causal model, whereas SEM commonly estimates latent variables built from the observed variables. Including fewer parameters also allowed for more parsimony with respect to model complexity, better leveraging our sample size.

## Results

### Descriptive statistics and zero-order correlations

First, we ran independent-samples *t*-tests to investigate gender differences in all study variables (see Table 1; significance level has been restricted to 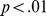 to account for multiple testing). Women scored significantly higher than men on agreeableness, conscientiousness, extraversion, openness to experience (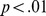), and marginally lower on emotional stability (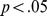). Women also reported significantly lower body esteem than men (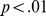) and the two groups reported similar levels of self-esteem and had a similar BMI (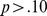). There were no order effects in men and women for any of the variables (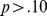). Due to the fact that gender interacts with most study variables (except for self-esteem and BMI), we present inter-correlations separately for men and women (Table 1). In both men and women, body esteem was positively associated with emotional stability, extraversion, self-esteem and negatively with BMI. In addition, body esteem was positively associated with conscientiousness in men and openness in women. Three personality traits, conscientiousness, emotional stability, and extraversion, were the strongest correlates of self-esteem in both genders. In addition, self-esteem was positively weakly associated with agreeableness and openness in women.

**Table 1:** Zero-order correlations and means (with standard deviations) of study variables in men (below the diagonal) and women (above the diagonal).

Variable	1	2	3	4	5	6	7	8	Women *M(SD)*	Men *M(SD)*
1. Agreeableness^1^	–	.07	.36***	.03	.18*	.17*	.14	−.14	4.89_a_(1.04)	4.58_b_(.96)
2. Conscientiousness^1^	−.07	–	.16*	.13	.19*	.22**	.14	−.12	5.36_a_(1.09)	4.73_b_(1.26)
3. Emotional stability^1^	.24**	.08	–	.23**	.13	.38***	.17*	−.08	4.56(1.22)	4.82(1.20)
4. Extraversion^1^	−.15	.14	.31***	–	.43***	.33***	.19*	.01	4.61_a_(1.29)	4.20_b_(1.30)
5. Openness^1^	.11	.13	.14	.35***	–	.18*	.19*	−.00	5.46_a_(.97)	5.10_b_(1.03)
6. Self-esteem^2^	.00	.26**	.36***	.35***	.16	–	.38***	−.10	19.98_a_(4.61)	20.58_a_(5.39)
7. Body esteem^3^	−.01	.18*	.28**	.29**	.14	.46***	–	−.33***	112.66_a_(20.77)	124.39_b_(5.39)
8. Body Mass Index^4^	−.09	−.11	−.16	−.02	.08	−.13	−.24***	–	24.21_a_(4.89)	24.05_a_(4.60)

*Notes.* Correlations *above* the diagonal are for women (*n* = 165) and values *below* the diagonal are for men (*n* = 133);

*p<.05,

**p<.01,

***p<.001.

Subscripts represent significant (*p*<.01, *df* = 296) gender differences across study variables (*t*-tests).

1 Ten-Item Personality Inventory (TIPI; [45]), scale 1–7, higher scores represent higher agreeableness, conscientiousness, etc.

2 Rosenberg Self-Esteem Scale (RSES; [49]), scale 0–30, higher score represents higher explicit self-esteem.

3 Body Esteem Scale (BES; [50]), scale 35–175, higher score represents higher overall body esteem.

4 Body Mass Index (BMI), usual range 15–40, higher score represents higher body mass.

Second, using one-way ANOVAs we investigated differences in study variables for Asian, Hispanic, and Caucasian American participants (see Table 2). The three racial/ethnic groups differed significantly in self-reported levels of self-esteem (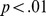), and only marginally in conscientiousness, extraversion, and overall body esteem (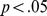). Post-hoc tests (Tukey HSD) revealed that Asian Americans had significantly lower self-esteem than Caucasian (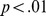) and marginally lower self-esteem than Hispanic Americans (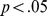). There were no significant differences across race/ethnicity in agreeableness, emotional stability, openness, and BMI.

**Table 2:** Means (with standard deviations) of study variables in Asian, Hispanic, and Caucasian participants.

Variable	Asian *M(SD)*	Hispanic *M(SD)*	Caucasian *M(SD)*
1. Agreeableness^1^	4.77(.91)	4.72(.97)	4.79(1.17)
2. Conscientiousness^1^	4.78(1.29)	5.28(1.04)	5.06(1.21)
3. Emotional stability^1^	4.58(1.09)	4.69(1.10)	4.76(1.48)
4. Extraversion^1^	4.14(1.44)	4.52(1.20)	4.58(1.31)
5. Openness^1^	5.10(1.05)	5.38(1.02)	5.38(.94)
6. Self-esteem^2^	18.90_a_(5.04)	20.47_a,b_(4.75)	21.28_b_(4.98)
7. Body esteem^3^	115.69(21.36)	116.39(22.99)	122.37(21.99)
8. Body Mass Index^4^	25.93(17.77)	24.63(4.77)	23.44(4.77)

*Notes.* Sample: Asian Americans (*n* = 86), Hispanic Americans (*n* = 127), Caucasian Americans (*n* = 85). Subscripts represent significant (*p*<.01, *df* = 2,297) racial/ethnic differences across study variables (ANOVAs).

1 Ten-Item Personality Inventory (TIPI; [45]), scale 1–7, higher scores represent higher agreeableness, conscientiousness, etc.

2 Rosenberg Self-Esteem Scale (RSES; [49]), scale 0–30, higher score represents higher explicit self-esteem.

3 Body Esteem Scale (BES; [50]), scale 35–175, higher score represents higher overall body esteem.

4 Body Mass Index (BMI), usual range 15–40, higher score represents higher body mass.

### Path analyses

A path analysis was used to test both hypothesis 1, stating that *self-esteem mediates the relationship between three personality traits (conscientiousness, emotional stability, extraversion) and body esteem,* and hypothesis 2, stating that *self-esteem does not mediate the relationship between the two remaining personality traits (agreeableness, openness) and body esteem.* Reverse mediational models, with body esteem mediating the relationship between personality and self-esteem, were also tested in this manner.

**Figure 1:**
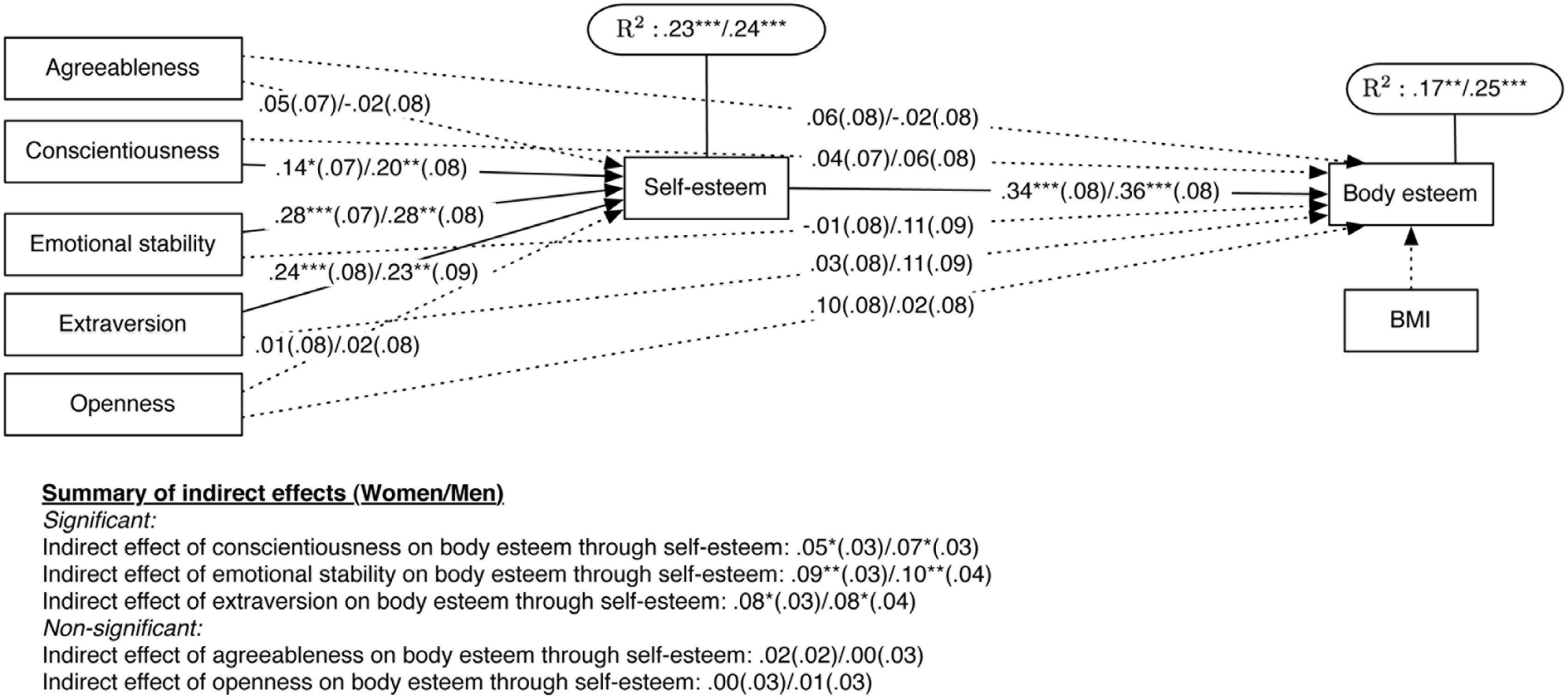
Path model presenting direct and indirect relationships between personality traits, self-esteem, and body esteem in women/men (paths include standardized regression coefficients with standard errors in brackets; each line presents first the coefficient for women and after a slash for men). *Note*: *p<.05, **p<.01, ***p<.001. Dotted lines represent paths with statistically non-significant coefficients.

**Figure 2:**
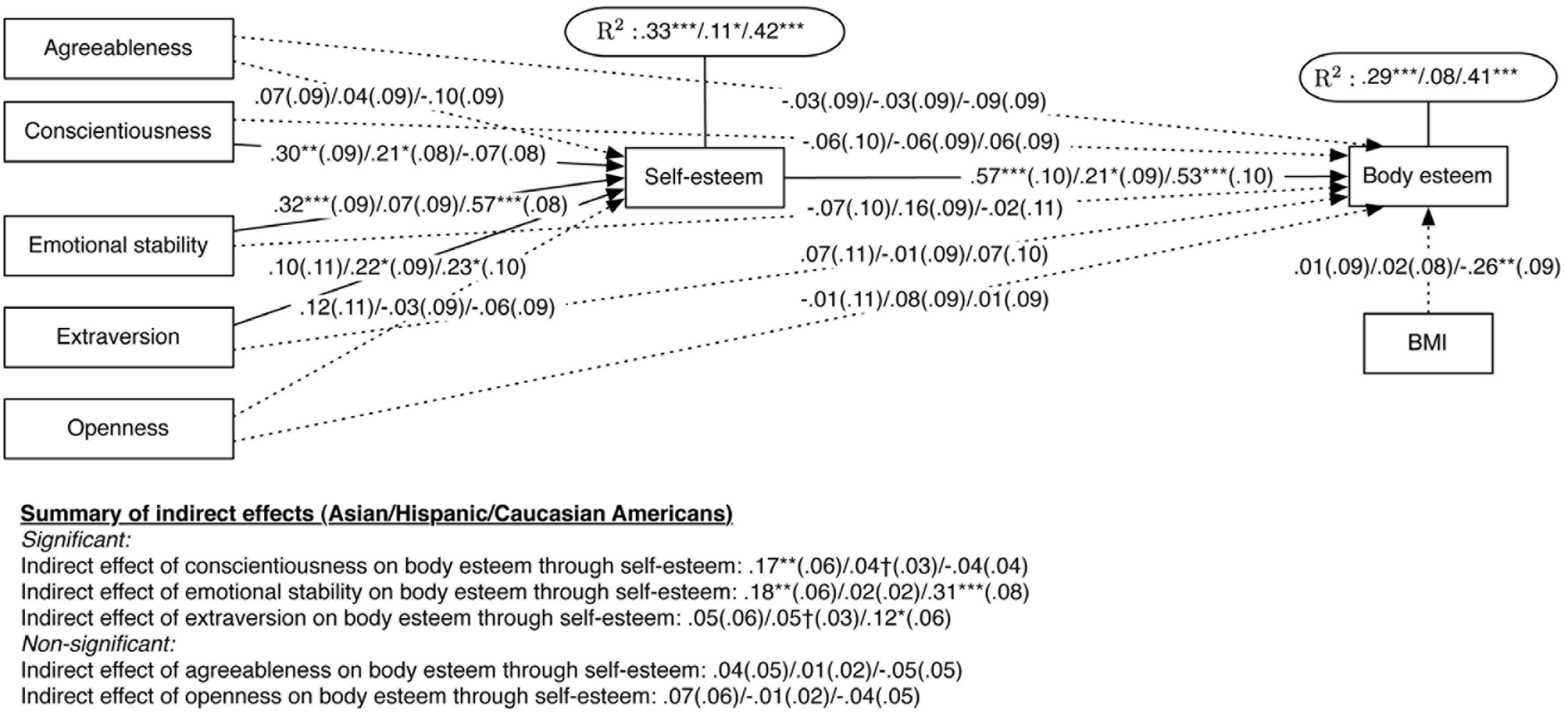
Path model presenting direct and indirect relationships between personality traits, self-esteem, and body esteem in Asian/Hispanic/Caucasian Americans (paths include standardized regression coefficients with standard errors in brackets; each line presents first the coefficient for Asian Americans, and after a slash for Hispanic and then Caucasian Americans). *Note*: *p<.05, **p<.01, ***p<.001, †*p*<.10. Dotted lines represent paths with statistically non-significant coefficients.

In the proposed model (see Figure 1 and Figure 2), all five personality traits (agreeableness, conscientiousness, emotional stability, extraversion, and openness) were specified as directly related to self-esteem and body esteem; self-esteem was directly related to body esteem, and BMI was included as a covariate of body esteem. In addition, we tested indirect effects between all personality traits and body esteem through self-esteem.

The results of the first set of path analyses are shown in Figure 1. The paths present standardized linear regression coefficients (with standard errors in brackets) for women and, after a slash, for men. The tested model adequately fit the data for both women (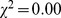, 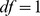, 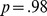; 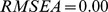; 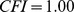; 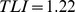) and men (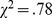, 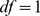, 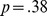; 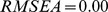; 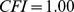; 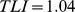) and the model fit was similar for men and women. A good fit was indicated by a non-significant chi-square, an RMSEA lower than.05, and CFI and TLI being close to 1. The CFI estimate was exactly 1.00 because there was only one degree of freedom and thus the fit was very good.

As presented in Figure 1, three personality traits were significantly related to self-esteem: conscientiousness, emotional stability, and extraversion. Self-esteem was the only significant predictor of body esteem. As evident from Figure 1, once self-esteem was included in the model, there were no significant direct relationships between any personality trait and body esteem. The indirect effects summarized at the bottom of the figure confirmed the mediational role of self-esteem by demonstrating that in both men and women three personality traits had significant *indirect* effects on body esteem through self-esteem: conscientiousness, emotional stability, and extraversion (women: 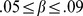, 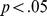; men: 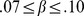, 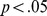). Figure 1 thus shows that in both men and women self-esteem is a pathway through which increased levels of conscientiousness, emotional stability, or extraversion increase body esteem. Consequently, hypothesis 1, stating that self-esteem would mediate the relationship between three personality traits (conscientiousness, emotional stability, and extraversion) and body esteem, was confirmed for both men and women.

Hypothesis 2, stating that self-esteem would *not* mediate the relationship between the remaining two personality traits (agreeableness, openness) and body esteem, was also confirmed in both genders, as there were no significant indirect effects between agreeableness, openness and body esteem through self-esteem (women: 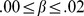, 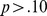; men: 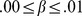, 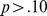).

The results for all racial/ethnic groups are shown in Figure 2. The paths present standardized linear regression coefficients (with standard errors in brackets) for Asian Americans, Hispanic Americans, and then Caucasian Americans. The proposed model adequately fit the data for Asian Americans (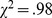, 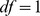, 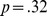; 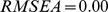; 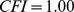; 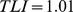), Hispanic Americans (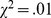, 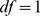, 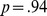;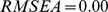; 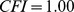; 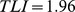), and Caucasian Americans (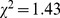, 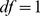, 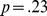;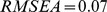; 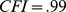; 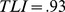); the model fit being slightly weaker for Hispanic Americans than the remaining two groups.

Figure 2 highlights slight differences in the proposed model for different racial/ethnic groups. In Asian American participants, conscientiousness and emotional stability (but not extraversion) were the only two personality traits significantly related to self-esteem and self-esteem was the only significant predictor of one’s body esteem. These two personality traits had significant indirect effects on body esteem through self-esteem (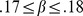, 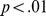). In Hispanic Americans, conscientiousness and extraversion (but not emotional stability) were significant predictors of self-esteem; self-esteem was the only significant predictor of one’s body esteem. These two personality traits had marginally significant indirect effects on body esteem through self-esteem (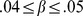, 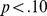). In Caucasian Americans, emotional stability and extraversion (but not conscientiousness) were significant predictors of self-esteem; both self-esteem (positively) and BMI (negatively) were significantly related to body esteem. Emotional stability and extraversion had significant indirect effects on body esteem through self-esteem (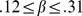, 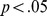). In other words, the results presented in Figure 2 suggest that, while self-esteem is a pathway through which personality traits affect body esteem, the specific personality traits differ across race/ethnicity (conscientiousness and emotional stability in Asian Americans; conscientiousness and extraversion in Hispanic Americans; emotional stability and extraversion in Caucasian Americans). Thus, hypothesis 1, stating that self-esteem would mediate the relationship between three personality traits (conscientiousness, emotional stability, and extraversion) and body esteem, was only partially confirmed for each of the racial/ethnic groups when they were considered separately. Hypothesis 2, stating that self-esteem would *not* mediate the relationship between the remaining two personality traits (agreeableness, openness) and body esteem, was confirmed in all groups, as there were no significant indirect effects between agreeableness, openness and body esteem through self-esteem (Asian: 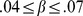, 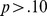; Hispanic: 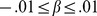, 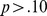; Caucasian: 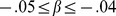, 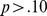).

#### A reverse model

To ensure that we are correctly specifying the direction of relationships between variables, we compared the model presented in Figure 1 with an alternative model representing the reverse mediational relationship, in which body esteem mediated the relationship between five personality traits and self-esteem. We ran two path analyses separately for women (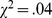, 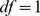,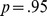;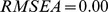; 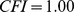; 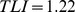) and men (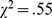,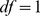, 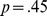; 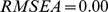; 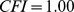; 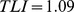). Even though both models fit well, there were no significant indirect effects for the relationships between personality traits and body esteem through self-esteem for either women (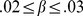, 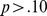) or men (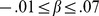, 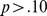), therefore, body esteem did not mediate the relationship between any of the personality trait and self-esteem in both men and women. This provides support for the directionality proposed above.

## Discussion

Prior work suggested that personality traits affect adolescents and young adults’ body esteem, but the exact nature and directionality of the relationship has not been specified. This study investigated the relationship between personality traits and body esteem in young men and women using path analyses. This work’s unique contributions include the elucidation of a mediational role for self-esteem that has not received much attention in the literature, as well as the exploration of these relationships across gender and race/ethnicity. The key to the identification of vulnerable populations at risk of developing eating disorders is a well-specified model of the relationship between personality traits, self-esteem, and body esteem. Our findings thus have relevance for the design of appropriate intervention programs for ethnically diverse adolescents of both genders.

The current research demonstrated that the relationship between personality traits and body esteem is more complex than shown in prior work [15], [16]. Rather than personality being directly related to body esteem, we found that the relationship between the three relevant personality traits (conscientiousness, emotional stability, and extraversion) and body esteem was mediated by self-esteem. This mediational relationship held for both men and women. In general, higher levels of conscientiousness, emotional stability, and extraversion were related to higher self-esteem, which in turn was related to higher body esteem. Once self-esteem was included in the model there were no significant direct relationships between personality traits and body esteem, suggesting full mediation. These results imply that individuals who score low on conscientiousness, emotional stability, and extraversion are at risk for lower self-esteem, which then inflates their risk of lower body esteem. We did not find the same mediational relationship for the two remaining Big Five personality traits (agreeableness and openness), which was not necessarily surprising, as they are less consistently related to both self-esteem and body esteem [22]; nonetheless, this pattern lends discriminant validity to our analytic approach.

While the mediational relationships were the same for men and women, several racial/ethnic differences were observed. In Asian American participants, self-esteem mediated the relationship between conscientiousness and body esteem and between emotional stability and body esteem, but not between extraversion and body esteem. In Hispanic Americans, self-esteem mediated the relationship between conscientiousness and body esteem and between extraversion and body esteem, but not between emotional stability and body esteem. Finally, in Caucasian Americans self-esteem was a mediator between emotional stability and body esteem and between extraversion and body esteem, but not between conscientiousness and body esteem. What can explain these differences? While a definitive answer must await future research, one possible explanation for the observed differences is the fact that culture shapes how people define themselves, and culture can thereby influence what factors relate to self-esteem (e.g., [54], [55]). Thus, it appears likely in light of our data that differences in the cultural construction of self-esteem appear in the groups we examined here. Racial/ethnic differences in the relationship between personality traits and body esteem also support the interaction between race/ethnicity and body esteem suggested in prior work [32], [56], pointing the way towards a promising avenue for future study.

The mediational mechanism involving self-esteem described in this study has clinical implications because it can used be used to design more effective self-esteem based intervention programs for young men and women displaying symptoms of eating disorders including low body esteem. Rather than focusing on body issues exclusively, our results suggest that efforts should also be made to address core issues of self-esteem more directly, since it sits in an intermediate position between personality traits and body esteem. Trait self-esteem is difficult to change and is partially hereditary (30% of variance is due to genetic variation; [57]). Nonetheless, there is evidence that self-esteem can be malleable, with prior work suggesting that it decreases slightly during a transition from elementary to junior high school but then it raises progressively through high school and college [58], [59]. Notably, the time around the transition from elementary and junior high school when adolescents are experiencing a decline in self-esteem is also the time when psychiatric disorders involving disordered eating increase in prevalence [60]. We suggest that this is the period during which interventions focusing on self-esteem (e.g., involving physical activity [61]) may be most effective, not only in terms of impacting self-esteem itself but also in the prevention of the potential downstream effects on body esteem and then disturbed eating. This parallels recommendations arising from a recent longitudinal study [62], which also stressed the importance of including self-esteem (together with stress and body importance) in body improvement intervention programs and demonstrated that self-esteem mediated a relationship between stress and body importance, which in turn predicted body dissatisfaction in adolescents. Moreover, the first major long-term controlled educational intervention focused on improving self-esteem was effective in preventing the development of eating disorders among Australian female adolescents [63]. This intervention improved the body satisfaction of female participants while helping them to avoid dieting and consequential weight loss observed in the female control group. A similar self-esteem based intervention has been successfully replicated among trainee health education and physical education teachers in Australia [64]. By contrast, other major randomized and controlled school-based interventions to prevent eating disorders among American, Australian, and Israeli adolescents employed an information-based strategy, which was ineffective [65]–[67]. This kind of information-based approach is among the most common intervention despite being one of the least effective, as shown by a recent meta-analysis of prevention programs among college students [68]. Indeed, several authors have warned that information-based preventive education efforts may do more harm than good because they introduce young men and women to dangerous methods of weight control and in some cases normalize or even glamorize eating disorders [69]–[71]. By linking eating disorder symptomology to self-esteem, our study provides support for the less frequently employed self-esteem based approach to the prevention of eating disorders during adolescence. More broadly, our findings support Bandura’s Social Learning Theory and Social Cognitive Theory [72], which theorizes that in order to change health behavior, individuals must first posses the required personal skills, perceptions, and self-efficacy to do so. Self-efficacy is a belief in one’s ability to achieve a goal, and is an ability that is strongly related to self-esteem.

We acknowledge a few limitations in the current approach. First, this research was conducted in an undergraduate sample rather than a representative sample of American adults. Therefore, we cannot generalize these findings to a general population.

Second, the data were collected using an online study, raising the possibility of disingenuous responses and a lack of control over the environment in which participants were taking surveys. However, web-based technology also has advantages in this domain. Research has shown that participants feel more comfortable with issues around confidentiality [73] and tend to be more honest [73], [74]. In addition, this methodology improves data quality by reducing data entry errors [75]. Further, several studies have found little systematic bias in computer-based surveys [74], [76], [77].

Third, the Ten-Item Personality Inventory (TIPI; [45]) used only two items to assess each personality trait; while recent data demonstrate that the TIPI meets standard psychometric standards [45], [78], one potential drawback of using a short personality inventory is that we are not able to examine relationships for lower order personality traits, which could improve specificity of our models.

Fourth, no behavioral measures (e.g., of eating behaviors) were included in the study. Such measures would increase the external validity of the work, and could fruitfully be included in future studies of this kind.

Finally, experimental manipulation or longitudinal data is needed to firmly establish temporal order between variables.

To conclude, the current study demonstrated that the relationship between personality traits and body esteem is more complex than previously realized. In particular, self-esteem mediates the relationship between three personality traits (conscientiousness, emotional stability, and extraversion) and body esteem. Future experimental and epidemiological research on body esteem, self-esteem and eating disorders should include all three of these constructs in order to accurately model the ways in which personality influences body image.
